# Erectile Dysfunction: Key Role of Cavernous Smooth Muscle Cells

**DOI:** 10.3389/fphar.2022.895044

**Published:** 2022-07-05

**Authors:** Iara Leão Luna de Souza, Elba dos Santos Ferreira, Luiz Henrique César Vasconcelos, Fabiana de Andrade Cavalcante, Bagnólia Araújo da Silva

**Affiliations:** ^1^ Departamento de Ciências Biológicas e da Saúde, Universidade Estadual de Roraima, Boa Vista, Brazil; ^2^ Programa de Pós-graduação em Produtos Naturais e Sintéticos Bioativos, Centro de Ciências da Saúde, Universidade Federal da Paraíba, João Pessoa, Brazil; ^3^ Departamento de Fisiologia e Patologia, Centro de Ciências da Saúde, Universidade Federal da Paraíba, João Pessoa, Brazil; ^4^ Departamento de Ciências Farmacêuticas, Centro de Ciências da Saúde, Universidade Federal da Paraíba, João Pessoa, Brazil

**Keywords:** smooth muscle, corpus cavernous, erection, flacity, authonomic nervous system, NANC, endothelin

## Abstract

Erectile dysfunction is increasingly affecting men, from the elderly to young adults, being a sexual disorder related to the inability to generate or maintain a penile erection. This disorder is related to psychosocial factors such as anxiety, depression, and low self-esteem, to organic factors such as the presence of preexisting conditions like hypertension, diabetes and dyslipidemia. The pathophysiology of the disease is related to changes in the neurotransmission of the autonomic or the non-cholinergic non-adrenergic nervous system, as well as the release of local mediators, such as thromboxane A_2_ and endothelin, and hormonal action. These changes lead to impaired relaxation of cavernous smooth muscle, which reduces local blood flow and impairs penile erection. Currently, therapy is based on oral vasodilation, such as sildenafil, tadalafil, vardenafil and iodenafil, or by direct administration of these agents into the corpus cavernosum or by intraurethral route, such as alprostadil and papaverine. Despite this, studies that consolidate the understanding of its pathophysiological process contribute to the discovery of new more efficient drugs for the treatment of erectile dysfunction. In this sense, in the present work an extensive survey was carried out of the mechanisms already consolidated and the most recent ones related to the development of erectile dysfunction.

## Introduction

Erectile dysfunction is characterized by the inability to achieve and/or maintain a suitable penile erection for a satisfactory sexual intercourse (NIH, 1993) and represents the sexual dysfunction most studied in men (Ückert et al., 2007). The first reports of this clinical disorder were found in ancient Egyptian writings dating back over 5,000 years, where reductions in both number and quality of erections of different Egyptians were described (Smith, 1974; Shah, 2002).

The international consultation committee for sexual medicine on definitions, epidemiology and risk factors for sexual dysfunction conducted an extensive analysis of the worldwide prevalence of erectile dysfunction. In this view, the prevalence of the disease was 1–10% in men under 40 years of age, 29% in those aged 40–49 years, 20–40% in men aged 60–69 years and 50–100% in men over 70 years of age (Nicolosi et al., 2003). In addition, the worldwide prevalence of erectile dysfunction is estimated at 322 million men by 2025 (Costa and Potempa, 2012).

Data regarding the erectile dysfunction incidence are less abundant. However, the number of new cases of the disease per year varies from 19 to 66 cases per 1,000 men, according to studies conducted in the United States, Netherlands, and Brazil (Johannes et al., 2000; Moreira et al., 2003; Schouten et al., 2005).

Erectile dysfunction is, predominantly, a vascular and benign disease, but it affects both physical and psychological health and has a significant impact on the quality of life of men and their partners, mainly due to the reduction of self-esteem and the commitment of the interpersonal relationship. The prevalence of the disease increases with age and can be seen as a serious public health problem (Medeiros-Júnior et al., 2014). The etiology is multifactorial, and, among the risk factors, the presence of cardiovascular diseases, sedentary lifestyle, smoking, diabetes, depression, anxiety and obesity are prominent (Alves et al., 2012).

Currently, erectile dysfunction is not limited to the reduction of sexual activity but acts as an indicator of systemic endothelial dysfunction. From the clinical point of view, this disease can precede cardiovascular events and can be used as an initial marker to identify men with high cardiovascular risk. In this case, patients with ED and no medical history of cardiovascular disease should be screened for cardiovascular disease (Gandaglia et al., 2014; Yafi et al., 2016; Hatzimouratidis et al., 2019).

Therefore, since alterations in the cellular signaling of cavernous smooth muscle cells can lead to this disease, this review focused on both contraction and relaxation processes that regulate penile erection, as well as highlights different targets as new approaches toward the development of new drugs for erectile dysfunction treatment.

## Physiological Control of Penile Erection

Penile erection is a neurovascular phenomenon modulated by psychological and hormonal factors, resulting in relaxation of cavernous smooth muscles from the penis. This phenomenon involves a complex interaction between the central nervous system and local stimuli. It is basically mediated by spinal reflexes, by processing information in the hypothalamus and by integrating tactile, visual, olfactory, auditory, and imaginary stimuli (Thomas, 2002).

Peripheral erection control depends on neuronal and local factors that ultimately influence the processes of cavernous smooth muscle contraction or relaxation. In this context, a muscle tone is generated and, thus, the functional state of the penis is maintained (Andersson, 2011).

Cavernous muscle tone modulation occurs through molecular mechanisms that depend on the action of agonists, such as neurotransmitters and endothelial-derived factors, and on the integrality of intracellular signaling. Specifically, an increase in intracellular Ca^2+^ concentration ([Ca^2+^]_i_) is the primary cause for the production of contraction, so the regulation of the intracellular levels of this ion and the sensitivity of contractile machinery are the key points for the regulation of smooth muscle cell (Maggi et al., 2000).

## Physiological Determinants of Penile Flaccidity

Sympathetic stimulation is the primary mechanism responsible for maintaining the penis in the flaccid state. In this case, noradrenaline (NA) released from noradrenergic neurons stimulates its receptors in penile vessels and cavernous smooth muscle cells from penis to induce contraction (Mills et al, 2001).

Pharmacological characterization of adrenergic receptors has shown that the expression of *a* subtype is 10 times more abundant than *ß* subtype in cavernous tissue. In addition, α_1A_, α_1B_, α_1D_, α_1L_ and α_2_ (α_2A-C_) adrenergic receptors are expressed in human corpus cavernosum (Maggi et al., 2000; Andersson, 2011). Interestingly, activation of all *a*-adrenergic receptor subtypes promotes smooth cavernous muscle contraction, since it was evidenced that presynaptic stimulation of α_2_-receptors inhibits non-adrenergic non-cholinergic transmission (NANC) (Filippi et al., 2002). Other important mediators of cavernous contraction include endothelin-1 (ET-1), prostaglandin F_2α_ (PGF_2α_), thromboxane A_2_ (TXA_2_) and angiotensin II (ANG II) (Tejada et al., 1991; Becker et al., 2001; Andersson, 2011).

**FIGURE 1:**
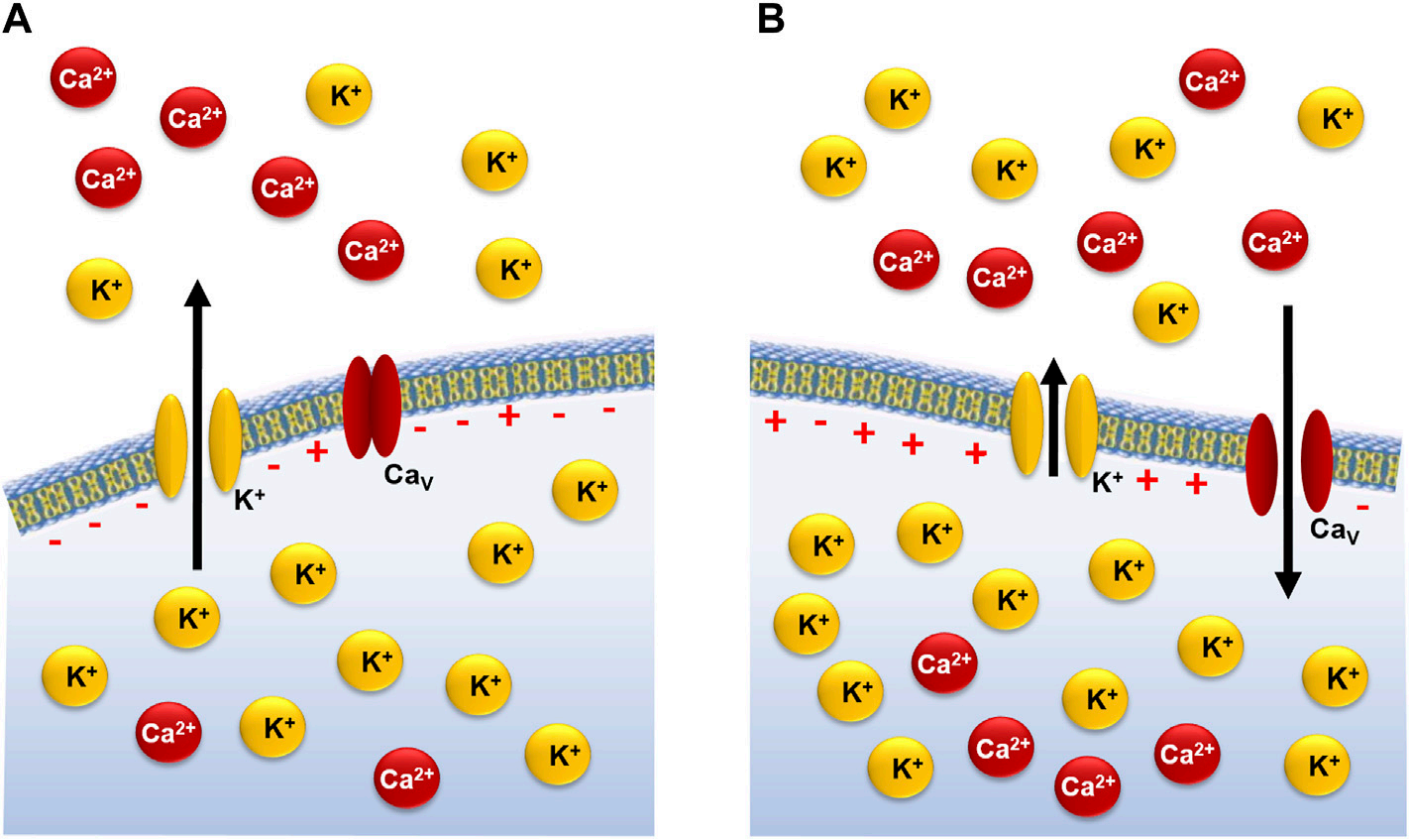
Electromechanical coupling of cavernous smooth muscle contraction during rest **(A)** and after increase in [K^+^]_e_ **(B)**.

In addition, the functional regulation of [Ca^2+^]_i_ to initiate a contractile response in the cavernous smooth muscle depends on two types of couplings: an electro- and a pharmacomechanical. The electromechanical coupling leads to the contractile response through membrane depolarization directly associated with increased extracellular K^+^ concentration ([K^+^]_e_) (Figure 1) or the action of K^+^ channel blockers (Rembold, 1996).

Despite the importance of electromechanical coupling of cavernous smooth muscle cell contraction, the condition of penile flaccidity arises mainly from the pharmacomechanical coupling triggered by the binding of agonists to G protein coupled receptors (GPCRs) and the activation of the inositol cascade through the G_q/11_. In this scenario, adrenaline, and NA binding to α_1_, ET-1 to ET_A/B_, PGF_2α_ to FP, TXA_2_ to TP and ANG II to AT_1_ receptors, which triggers the activation of phospholipase C_β1_ (PLC_β1_) pathway. In this signaling, PLC_β1_ hydrolyzes the phosphatidylinositol 4,5-bisphosphate (PIP_2_), producing inositol 1,4,5-trisphosphate (IP_3_) and diacylglycerol (DAG) (Berridge, 2008). IP_3_ induces Ca^2+^ release from the sarcoplasmic reticulum (SR) by activating the IP_3_ receptors (IP_3_R). In addition, in SR are expressed ryanodine receptors (RyR), caffeine sensitive Ca^2+^ release channels, which are activated by Ca^2+^ previously released via IP_3_R in a process called Ca^2+^-induced Ca^2+^ release (CICR) (Dellis et al., 2006; Mchale et al., 2006).

**FIGURE 2:**
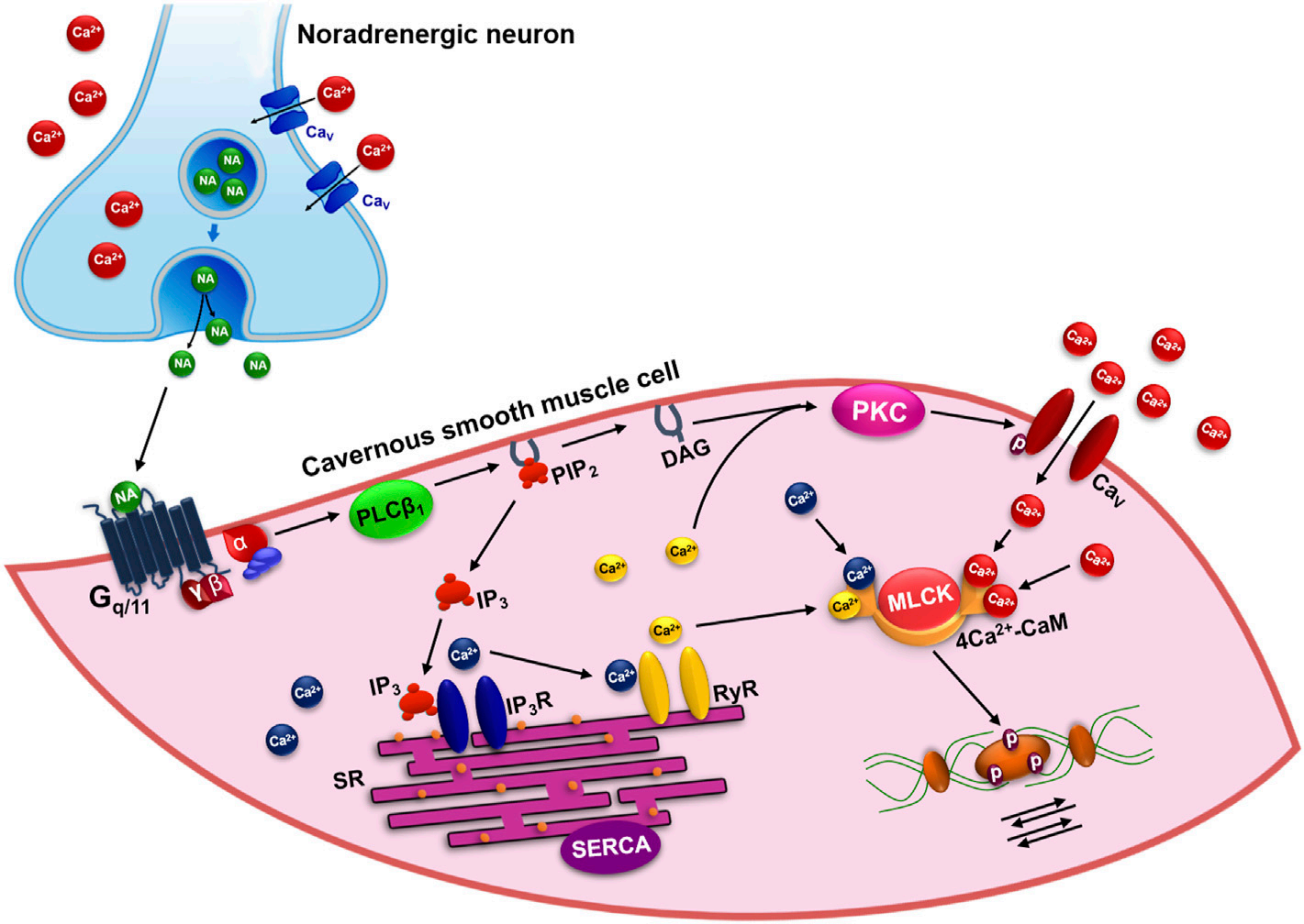
Pharmacomechanical mechanism of contraction in the cavernous smooth muscle by activation of G_q/11_-PLCβ_1_ pathway. NA: noradrenaline; Ca_V_: voltage-dependent Ca^2+^ channels; PLCβ_1_: phospholipase Cβ_1_; PIP_2_: phosphatidylinositol 4,5-bisphosphate; DAG: diacylglycerol; IP_3_: inositol 1,4,5-trisphosphate; IP_3_R: IP_3_ receptors; RyR: ryanodine receptors; SR: sarcoplasmic reticulum; SERCA: Ca^2+^-ATPase of SR; PKC: Ca^2+^-dependent protein kinase; MLCK: myosin light chain kinase; CaM: calmodulin protein.

Following this signaling cascade, Ca^2+^ and DAG activate the Ca^2+^ dependent protein kinase (PKC), which, in turn, leads to [Ca^2+^]_i_ increase through phosphorylation and the direct activation of the voltage-dependent Ca^2+^ channels (CaV) present in plasma membrane (Fukata et al, 2001). The increase in [Ca^2+^]_i_ favors the interaction of this ion with calmodulin protein (CaM), leading to 4Ca^2+^-CaM complex formation. This complex activates myosin light chain kinase (MLCK), which phosphorylates the regulatory myosin light chain (rMLC), promoting the interaction of myosin and actin filaments, triggering the process of contraction of the cavernous smooth muscle (Figure 2) (Webb, 2003).

**FIGURE 3:**
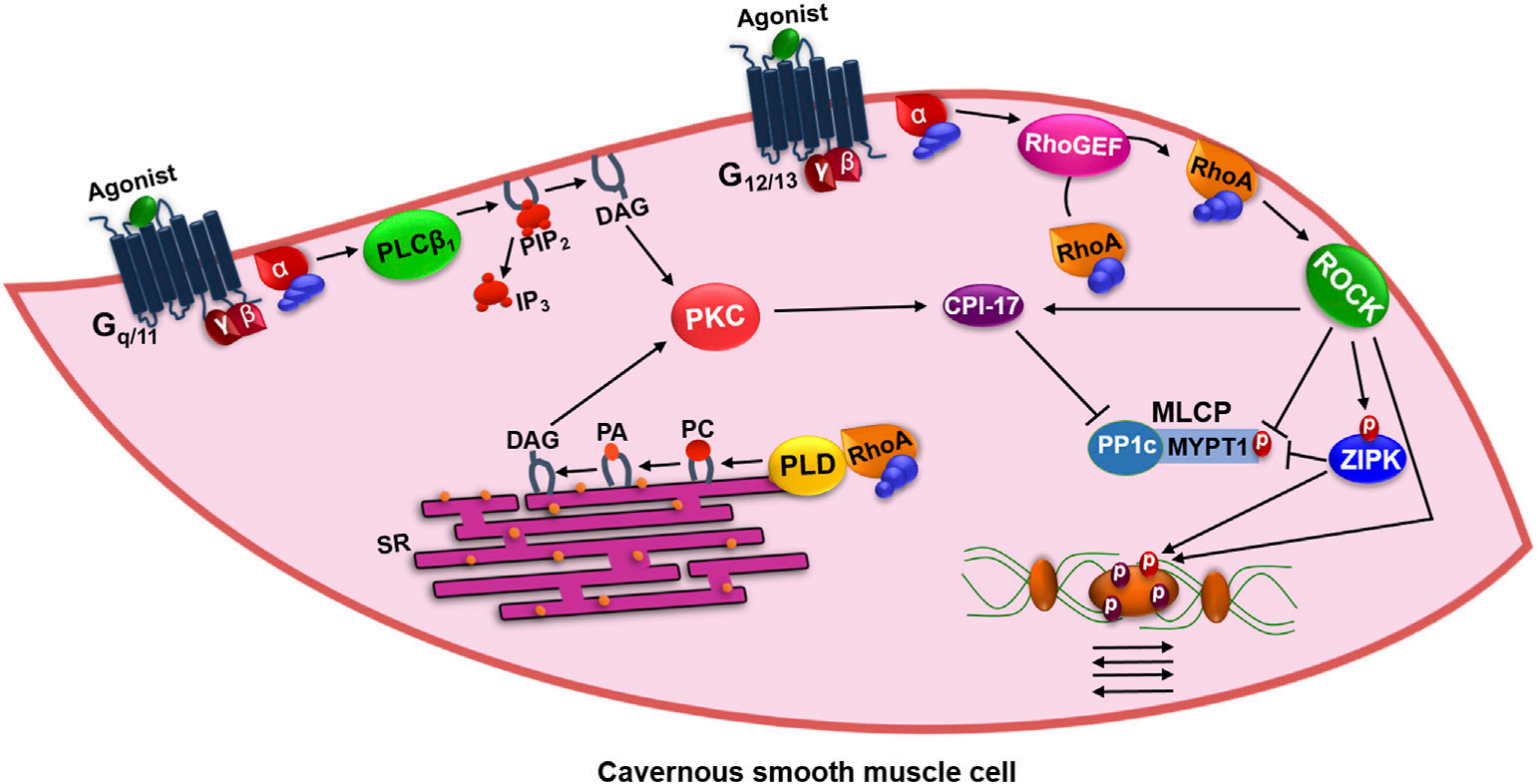
Mechanism of maintenance of contraction in the cavernous smooth muscle by activation of G_12/13_/ROCK pathway. PLCβ_1_: phospholipase Cβ_1_; PIP_2_: phosphatidylinositol 4,5-bisphosphate; DAG: diacylglycerol; IP_3_: inositol 1,4,5-trisphosphate; SR: sarcoplasmic reticulum; PKC: Ca^2+^-dependent protein kinase; RhoA: small GTP binding protein G; PLD: phospholipase D; PC: phosphatidylcholine; PA: phosphatidic acid; RhoGEF: RhoA guanine nucleotide exchange factor; ROCK: kinase of RhoA; CPI-17: PKC-dependent phosphatase inhibitor of 17 kDa; ZIPK: zipper-interacting protein kinase; MLCP: myosin light chain phosphatase; MYPT1: regulatory subunit of MLCP; PP1c: catalytic subunit of MLCP.

Although [Ca^2+^]_i_ increase is transient, cavernous smooth muscle cells are able to maintain the contracted state even after the reduction of the intracellular levels of this ion. In fact, studies have reported an alternative pathway that contributes to the maintenance of smooth muscle contraction, designated as a Ca^2+^ sensitization pathway (Figure 3). This pathway involves the modulation of myosin light chain phosphatase (MLCP), mainly by the small GTP binding protein G (RhoA) and its associated kinase (ROCK), a serine/threonine kinase (Hori and Karaki, 1998). In the inactivated state, RhoA is bound to guanosine diphosphate (GDP) and the guanine dissociation inhibitory protein (RhoGDI), forming a complex found in cytoplasm (Jin and Burnett, 2006).

Several contractile agonists, such as ET-1 and ANG II, which normally increase [Ca^2+^]_i_ via GPCRs lead to the direct activation of the RhoA guanine nucleotide exchange factor (RhoGEF) by the G_12/13_ proteins, which activate RhoA by exchanging GDP for guanosine triphosphate (GTP) in this protein (Somlyo and Somlyo, 2003). Thus, RhoA-GTP is translocated to plasma membrane, where it remains anchored by geranilgeranilization, and activates the ROCK. The activity of RhoA is reduced by the action of Rho-GTPase activating protein (RhoGAP), which culminates with the GTP cleavage in GDP and the formation of the inactive RhoA-GDP/RhoGDI complex, returning to cytoplasm (Burridge and Wennerberg, 2004).

After activation by RhoA-GTP, ROCK directly phosphorylates rMLC and the regulatory subunit MYPT1 of MLCP, making it inactive, promoting the maintenance of the phosphorylated state of rMLC and, consequently, the contraction force. In addition, it can activate a Ca^2+^ dependent kinase protein, also known as zipper-interacting protein kinase (ZIPK). The ZIPK phosphorylates directly the rMLC, however its main target is the Thr^696^ residue of MYPT1 which, when phosphorylated, inhibits the action of MLCP (Murthy, 2006).

CPI-17 (PKC-dependent phosphatase inhibitor of 17 kDa) represents a substrate for both ROCK and PKC. When phosphorylated, CPI-17 binds to the PP1c catalytic subunit of MLCP to inhibit its enzymatic activity and prolong smooth muscle contraction (Kitazawa et al., 2000).

RhoA-GTP also stimulates phospholipase D (PLD), this enzyme is mainly associated with intracellular membranes, but is also found in the plasma membrane and is specific for phosphatidylcholine (PC), releasing phosphatidic acid (PA) that through the action of phosphohydrolase enzyme is dephosphorylated to DAG leading to sustained activation of PKC. Activation of PKC may also be dependent on G_q/11_-PLCβ1, which forms DAG by PIP_2_ hydrolysis. PKC can phosphorylate the Thr^38^ residue of CPI-17, thereby increasing its inhibitory potency over PP1c by more than 1000-fold, inhibiting the action of MLCP (Murthy, 2006; Berridge, 2009).

Mitogen-activated protein kinases (MAPK) have been associated with increased smooth muscle contractility, proliferation, and chemotaxis. Among them, MAPK ERK1/2 and p38 are especially noteworthy, especially for their activity on vascular smooth muscle, however, molecular mechanisms that directly alter cavernous contractility are not fully understood (Berridge, 2009).

## Physiological Determinants of Penile Erection

Relaxation of penile corpus cavernosum occurs in response to NANC and cholinergic neurotransmission, with nitric oxide (NO) as the most important neurotransmitter. The synthesis of NO is catalyzed by nitric oxide synthase (NOS) enzyme that in the presence of O_2_ converts l-arginine to l-citrulline and NO in both endothelial cells and nerve endings. Three distinct isoforms of NOS are expressed in human cavernous smooth muscle cells, the constitutive and calcium dependent isoforms include neuronal (nNOS) and endothelial (eNOS). In addition, the inducible isoform (iNOS) was identified, especially when there is tissue injury (Burnett et al., 1993; Gonzalez-Cadavid et al, 1999).

In endothelial cells, eNOS activation may occur in response to shear stress induced by increased blood flow in the penile vessels, leading to phosphatidylinositol 3-kinase (PI3K) activation, which phosphorylates PIP_2_ and forms the 3,4,5-phosphatidylinositol trisphosphate (PIP_3_). PIP_3_ recruits and activates phosphoinositide-dependent kinase-1 (PDK-1) that, in turn, phosphorylates and activates Akt, also known as protein kinase B (PKB). Akt is a serine/threonine kinase that phosphorylates directly the eNOS on its Ser^1177^ residues, resulting in increased activity of this enzyme, a phenomenon that contributes to continuous NO production (Hurt et al., 2002; Sommer et al., 2002).

Other substances are responsible for modulating cavernous smooth muscle relaxation, such as vasoactive intestinal peptide (VIP) and calcitonin gene related peptide (CGRP), released by cholinergic nerve endings. The endothelium also releases relaxing prostanoids, such as prostacyclin (PGI_2_) and prostaglandins E types 1 and 2 (PGE_1_ and PGE_2_) (Molderings et al., 1992; Porst, 1996).

**FIGURE 4:**
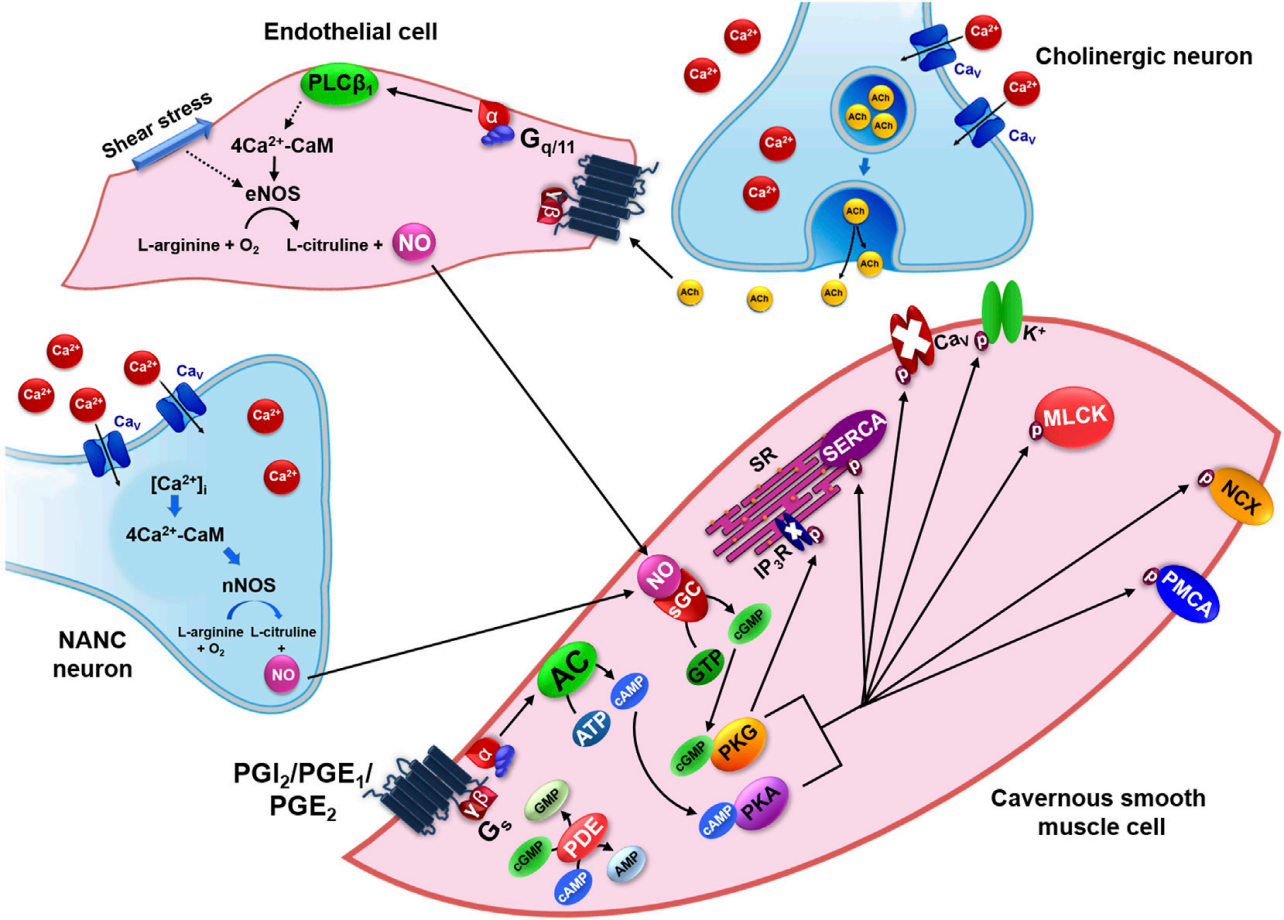
Pharmacomechanical mechanism of relaxation in the cavernous smooth muscle by activation of NO-sGC-PKG and G_s_-AC-PKA pathways. NANC: non-adrenergic non-cholinergic transmission; [Ca^2+^]_i_: intracellular Ca^2+^ concentration; CaM: calmodulin protein; nNOS: neuronal nitric oxide synthase; NO: nitric oxide; Ca_V_: voltage-dependent Ca^2+^ channels; eNOS: endothelial nitric oxide synthase; PGI_2_: prostacyclin; PGE_1/2_: prostaglandins E types 1 and 2; AC: adenylyl cyclase; ATP: adenosine triphosphate; cAMP: cyclic adenosine monophosphate; PKA: cAMP-dependent protein kinase; AMP: adenosine monophosphate; sGC: soluble guanylyl cyclase receptor; GTP: guanosine triphosphate; cGMP: cyclic guanosine monophosphate; PKG: cGMP-dependent protein kinase; GMP: guanosine monophosphate; IP_3_: inositol 1,4,5-trisphosphate; SR: sarcoplasmic reticulum; SERCA: Ca^2+^-ATPase of sarcoplasmic reticulum; Ca_V_: voltage-dependent Ca^2+^ channels; MLCK: myosin light chain kinase; NCX: Na^+^/Ca^2+^ exchanger; PMCA: Ca^2+^-ATPase of plasma membrane; PDE: phosphodiesterase enzyme.

Although these substances are involved in cavernous smooth muscle relaxation, NO is considered the most important physiological mediator for penile erection. When there is a sexual stimulus, it is released by NANC neurons or endothelial cells in response to ACh and, as a soluble gas; it diffuses and activates its soluble guanylyl cyclase receptor (cGC) in cavernous smooth muscle cell (Figure 4) (Andersson, 2011).

The cGC converts GTP into cyclic guanosine monophosphate (cGMP) that activates cGMP-dependent protein kinase (PKG). This kinase phosphorylates several substrates to promote cavernous muscle relaxation (Figure 4). These include: 1) activation of K^+^ channels that indirectly, by repolarization or hyperpolarization, can inhibit Ca_V_; 2) direct inhibition of Ca_V_, decreasing Ca^2+^ influx (Rembold, 1996); 3) increase in Ca^2+^-ATPase kinetics of both SR (SERCA) and plasma membrane (PMCA), thus increasing Ca^2+^ sequestration and extrusion, respectively; 4) decrease of [Ca^2+^]_i_ by Na^+^/Ca^2+^ exchanger (NCX) activation that acts in a ratio of 3/1 (Blaustein, 1989); 5) inhibition of MLCK, reducing its affinity to 4Ca^2+^-CaM complex, preventing the phosphorylation of rMLC and, consequently, the contractile process (Rembold, 1996); 6) inactivation of IP_3_R that reduces Ca^2+^ release from RS (Woodrum and Brophy, 2001); 7) RhoA inactivation by phosphorylation of Ser^188^ residue, displacing it into the cytosol; 8) MLCP activation by phosphorylation of Ser^695^ residue from MYPT1 subunit which prevents phosphorylation of Thr^696^ residue by ROCK; 9) MLCP activation by telokin phosphorylation (endogenous activator of MLCP), being an independent mechanism of RhoA (Murthy, 2006).

From the physiological point of view, NO derived from nNOS is responsible for the onset of erection, whereas NO produced by eNOS in response to shear stress contributes to the maintenance of penile rigidity during erection (Andersson, 2011).

The other relaxing mediators, such as prostaglandins E_1-2_ and prostacyclin act on EP_2_, EP_4_ and IP receptors, respectively, all coupled to Gs protein. The *a*-GTP subunit activates adenylyl cyclase (AC), which converts adenosine triphosphate (ATP) into cyclic adenosine monophosphate (cAMP), responsible for activating cAMP-dependent protein kinase (PKA). This kinase phosphorylates the same targets of PKG to promote cavernous muscle relaxation, with the exception of IP_3_R (Figure 4) (Andersson, 2011).

Due to cGMP and cAMP importance in [Ca^2+^]_i_ reduction, highlights the essential role for the regulation of erectile function performed by the phosphodiesterases enzymes (PDEs), which hydrolyze cGMP and cAMP to their non-cyclic forms (GMP and AMP, respectively) and enclose the signaling cascade that results in cavernous smooth muscle relaxation. Each PDE family includes multiple isoforms that can act on a specific cyclic nucleotide or on both types. In penis, the predominant phosphodiesterase isoform is 5 (PDE5), which acts specifically on cGMP (Mancina et al., 2005).

As the balance between the contraction and relaxation processes of the cavernous smooth muscle regulates the processes of penile flaccidity and erection, any deregulation in steps of these signaling pathways may compromise penile function and thus contribute to erectile dysfunction development.

## Cavernous Smooth Muscle Cells as Targets for the Pharmacological Treatment of Erectile Dysfunction

### Intracavernous and Intraurethral Therapies

Currently, they represent the last line of treatment and show, as advantages, speed to start, less than 10 min, and quality of penile erections, even in the absence of sexual stimulation. In this case, the man needs to receive previous training to be able to apply the intracavernous injection or to position the intraurethral suppository. The vasodilatory substances commonly used to induce penile erection are alprostadil, papaverine and phentolamine alone or in combinations of two or three of them with high success rates therapeutic use (90%) (Hatzimouratidis and Hatzichristou, 2005; Hatzimouratidis et al., 2019).

Alprostadil is the synthetic prostaglandin E_1_, thus, when it binds to EP_2/4_ receptors it activates the AC signaling pathway, culminating in an increase in the intracellular concentration of cAMP ([cAMP]_i_), which through previously described mechanisms triggers corpus cavernosum relaxation and, consequently, penile erection. It is used in intracavernous injection therapy and as a suppository for intraurethral use (Moreland et al., 2003).

Papaverine non-selectively inhibits phosphodiesterases and thus increases [cAMP]_i_ and the intracellular concentration of cGMP ([cGMP]_i_), with similar results to that seen with alprostadil, however, it is only used in intracavernous injection therapy. In addition to these substances, selective α_1_-adrenoceptor antagonist phentolamine prevents vasoconstriction resulting from the activation of the PLCβ_1_ pathway and thereby assists penile erection. It is used only in intracavernous injection therapy (Virag et al., 1991).

The main risk of using these drugs is priapism, which consists of a painful and prolonged penile erection (longer than 2 hours), independent of sexual desire and due to insufficient penile blood drainage. In cases of priapism, emergency medical attention is indicated in order to perform blood aspiration of the corpus cavernosum, the application of phenylephrine, a selective α_1-_adrenergic agonist, which promotes local vasoconstriction through the activation of the PLCβ_1_ pathway and, in more severe cases, the creation of shunts between the corpus cavernosum and the glans or spongy body through surgical procedures (Linet and Ogrinc, 1996).

### Oral Therapy

Primary pharmacotherapy for erectile dysfunction treatment involves the use of PDE5 inhibitors, such as sildenafil, which is the prototype of the group, tadalafil, vardenafil and iodenafil. Mechanically, PDE5 inhibitors increase [cGMP]_i_ and thus initiate the cascade of intracellular events that result in relaxation of the corpus cavernosum and promote penile erection. However, previous sexual stimulation is essential to increase intracellular NO levels and, consequently, to generate cGMP (Yafi et al., 2016).

PDE5 inhibitor use improves the sexual performance of the men, without alteration of the libido. Recently, the discovery that the use of these inhibitors reduces the refractory period, a time when a temporary physiological flaccidity occurs immediately after ejaculation, has promoted an increase in the demand for these drugs by young and potent men (Ekmekçioğlu et al., 2005; Hatzimouratidis et al., 2019).

Despite the great therapeutic success of these drugs, about 30–40% of the men affected by erectile dysfunction do not respond to this first line of treatment. Additionally, PDE5 inhibitor use should be performed with caution in patients with cardiovascular impairment, such as uncontrolled hypertension and unstable angina. In addition, the use of these drugs is contraindicated in patients who use nitrates because of the increased risk of severe hypotension (Eardley and Sethia, 2003).

The major side effects related to therapy with PDE5 inhibitors include headache, nasal congestion, and facial flushing. In addition, visual and auditory changes, including macular degeneration, anterior ischemic optic neuropathy, hearing loss and tinnitus are recent reasons for precautions. These complications appear to be related to the mild inhibitory action of these drugs on PDE6 (Zelefsky et al., 2014; Hatzimouratidis et al., 2019).

### Targets Studied as an Alternative to the Use of Oral Therapy

Despite the use of oral therapy being the first line of treatment for erectile dysfunction, there are patients who are refractory to treatment, creating a field of research for new drugs. In this sense, there are different targets in the contractile machinery of the penile corpus cavernosum.

In clinical trials, the use of agents that act as activators of the soluble guanylyl cyclase enzyme have been shown to improve the development of penile erection. Additionally, improvement in erectile dysfunction was also observed with the therapeutic use of Rho-kinase inhibitors. However, in terms of clinical efficacy and adverse effect profile, there was no superiority when compared to PDE inhibitors (Sanofi-Avenis, 2010; Bayer, 2017).

Another approach considered in the function of cavernous smooth muscle cells is to promote the opening of potassium channels, which would lead to a reduction in the intracellular concentration of calcium and, consequently, to relaxation. In this sense, the use of calcium-sensitive Maxi-K ion channel gene (hSlo cDNA) was evaluated in clinical phase I studies, demonstrating a good safety of use, however, there was no improvement to the use of PDE inhibitors, limiting clinical progress (Melman, 2006).

## Conclusion

Balance among flaccidity and penile erection depends on the timing between the contraction and relaxation processes of cavernous smooth muscle cells. Thus, [Ca^2+^]_i_ is the central point of regulation of smooth muscle tone. In this view, the increase in [Ca^2+^]_i_ leads to contraction of these cavernous cells and, consequently, maintains the penis in the flaccid state, while relaxation, which promotes penile erection, is mediated by the reduction of [Ca^2+^]_i_. In this sense, the imbalance of these mechanisms is related to ED development.

Currently, the main therapeutic lines for ED present as targets steps of contraction and relaxation signaling in cavernous smooth muscle cells. Therefore, further research targeting different components of electro- and pharmacomechanical couplings of cavernous smooth muscle cell arise as new targets for the development of promising drugs for the treatment of this disease.

## References

[B1] Alves M. A. Queiroz T. M. Medeiros I. A. (2012). Fisiologia peniana e disfunção erétil: uma revisão de literatura. Rev. Bras. Ciênc. Saúde 16, 439–444. 10.4034/rbcs.2012.16.03.23

[B2] Andersson K. E. (2011). Mechanisms of Penile Erection and Basis for Pharmacological Treatment of Erectile Dysfunction. Pharmacol. Rev. 63, 811–859. 10.1124/pr.111.004515 21880989

[B3] Bayer Healthcare A. G. (2017). A Prospective, Randomized, Doubleblind, Double-Dummy, Placebo and Active Controlled, Multicenter Study Assessing the Efficacy and Safety of the Combination BAY 604552/vardenafil Compared to Vardenafil (20 Mg) for the Treatment of Erectile Dysfunction Not Sufficiently Responsive to Standard Therapy with PDE5 Inhibitors. Bethesda (MD): National Library of Medicine (US), ClinicalTrials.gov.

[B4] Becker A. J. Uckert S. Stief C. G. Truss M. C. Hartmann U. Jonas U. (2001). Systemic and Cavernosal Plasma Levels of Endothelin (1-21) during Different Penile Conditions in Healthy Males and Patients with Erectile Dysfunction. World J. Urol. 19, 371–376. 10.1007/s003450100213 11760787

[B5] Berridge M. J. (20082008). Smooth Muscle Cell Calcium Activation Mechanisms. J. Physiol. 586, 5047–5061. 10.1113/jphysiol.2008.160440 PMC2652144 18787034

[B6] Berridge M. J. (2009). “Cell Signalling Pathways,” in Cell Signalling Biology (Portland Press Limited), 1–118.

[B7] Blaustein M. P. (1989). “Chapter 15 Sodium-Calcium Exchange in Cardiac, Smooth, and Skeletal Muscles: Key to Control of Contractility,” in Current Topics in Membranes and Transport. Editors Hoffman J. F. Glebisch G. (San Diego: Academic Press), 289–330. 10.1016/s0070-2161(08)60019-2

[B8] Burnett A. L. Tillman S. L. Chang T. S. Epstein J. I. Lowenstein C. J. Bredt D. S. (1993). Immunohistochemical Localization of Nitric Oxide Synthase in the Autonomic Innervation of the Human Penis. J. Urol. 150, 73–76. 10.1016/s0022-5347(17)35401-0 7685426

[B9] Burridge K. Wennerberg K. (2004). Rho and Rac Take center Stage. Cell 116, 167–179. 10.1016/s0092-8674(04)00003-0 14744429

[B10] Costa P. Potempa A. J. (2012). Intraurethral Alprostadil for Erectile Dysfunction: a Review of the Literature. Drugs 72, 2243–2254. 10.2165/11641380-000000000-00000 23170913

[B11] Dellis O. Dedos S. G. Tovey S. C. Taufiq-Ur-Rahman Dubel S. J. Taylor C. W. (2006). Ca2+ Entry through Plasma Membrane IP3 Receptors. Science 313, 229–233. 10.1126/science.1125203 16840702

[B12] Eardley I. Sethia K. (2003). Erectile Dysfunction: Current Investigation and Management. Elsevier Health Sciences.

[B13] Ekmekçioğlu O. Inci M. Demirci D. Tatlişen A. (2005). Effects of Sildenafil Citrate on Ejaculation Latency, Detumescence Time, and Refractory Period: Placebo-Controlled, Double-Blind, Crossover Laboratory Setting Study. Urology 65, 347. 15708051 10.1016/j.urology.2004.09.012

[B14] Filippi S. Luconi M. Granchi S. Natali A. Tozzi P. Forti G. (2002). Endothelium-dependency of Yohimbine-Induced Corpus Cavernosum Relaxation. Int. J. Impot. Res. 14, 295–307. 10.1038/sj.ijir.3900890 12152120

[B15] Fukata Y. Amano M. Kaibuchi K. (2001). Rho-Rho-kinase Pathway in Smooth Muscle Contraction and Cytoskeletal Reorganization of Non-muscle Cells. Trends Pharmacol. Sci. 22, 32–39. 10.1016/s0165-6147(00)01596-0 11165670

[B16] Gandaglia G. Briganti A. Montorsi F. Vlachopoulos C. (2014). Reply to Christopher Chee Kong Ho, Siew Eng Ho, Srijit Das' letter to the editor re: Giorgio Gandaglia, Alberto Briganti, Graham Jackson, et al. A systematic review of the association between erectile dysfunction and cardiovascular disease. Eur Urol 2014;65:968-78. Eur. Urol. 66, e88–9. 10.1016/j.eururo.2014.06.004 24951362

[B17] Gonzalez-Cadavid N. F. Ignarro L. J. Rajfer J. (1999). Nitric Oxide and the Cyclic GMP System in the Penis. Mol. Urol. 3, 51–59. 10851306

[B18] Hatzimouratidis K. Hatzichristou D. G. (2005). A Comparative Review of the Options for Treatment of Erectile Dysfunction: Which Treatment for Which Patient? Drugs 65, 1621–1650. 10.2165/00003495-200565120-00003 16060698

[B19] Hatzimouratidis K. Giuliano F. Moncada I. Muneer A. Salonia A. Verze P. (2019). EAU Guidelines on Erectile Dysfunction, Premature Ejaculation, Penile Curvature and Priapism, 1–95.

[B20] Hori M. Karaki H. (1998). Regulatory Mechanisms of Calcium Sensitization of Contractile Elements in Smooth Muscle. Life Sci. 62, 1629–1633. 10.1016/s0024-3205(98)00119-2 9585148

[B21] Hurt K. J. Musicki B. Palese M. A. Crone J. K. Becker R. E. Moriarity J. L. (2002). Akt-dependent Phosphorylation of Endothelial Nitric-Oxide Synthase Mediates Penile Erection. Proc. Natl. Acad. Sci. U S A. 99, 4061–4066. 10.1073/pnas.052712499 11904450 PMC122648

[B22] Jin L. Burnett A. L. (2006). RhoA/Rho-kinase in Erectile Tissue: Mechanisms of Disease and Therapeutic Insights. Clin. Sci. (Lond) 110, 153–165. 10.1042/CS20050255 16411892

[B23] Johannes C. B. Araujo A. B. Feldman H. A. Derby C. A. Kleinman K. P. McKinlay J. B. (2000). Incidence of Erectile Dysfunction in Men 40 to 69 Years Old: Longitudinal Results from the Massachusetts Male Aging Study. J. Urol. 163, 460–463. 10.1016/s0022-5347(05)67900-1 10647654

[B24] Kitazawa T. Eto M. Woodsome T. P. Brautigan D. L. (2000). Agonists Trigger G Protein-Mediated Activation of the CPI-17 Inhibitor Phosphoprotein of Myosin Light Chain Phosphatase to Enhance Vascular Smooth Muscle Contractility. J. Biol. Chem. 275, 9897–9900. 10.1074/jbc.275.14.9897 10744661

[B25] Linet O. I. Ogrinc F. G. (1996). Efficacy and Safety of Intracavernosal Alprostadil in Men with Erectile Dysfunction. The Alprostadil Study Group. N. Engl. J. Med. 334, 873–877. 10.1056/NEJM199604043341401 8596569

[B26] Maggi M. Filippi S. Ledda F. Magini A. Forti G. (2000). Erectile Dysfunction: from Biochemical Pharmacology to Advances in Medical Therapy. Eur. J. Endocrinol. 143, 143–154. 10.1530/eje.0.1430143 10913932

[B27] Mancina R. Filippi S. Marini M. Morelli A. Vignozzi L. Salonia A. (2005). Expression and Functional Activity of Phosphodiesterase Type 5 in Human and Rabbit Vas Deferens. Mol. Hum. Reprod. 11, 107–115. 10.1093/molehr/gah143 15640438

[B28] Mchale N. Hollywood M. Sergeant G. Thornbury K. (2006). Origin of Spontaneous Rhythmicity in Smooth Muscle. J. Physiol. 570, 23–28. 10.1113/jphysiol.2005.098376 16239271 PMC1464298

[B29] Medeiros Júnior J. L. Oliveira F. A. Silva P. C. Furriel A. Sampaio F. J. Gregório B. M. (2014). Lard And/or Canola Oil-Rich Diets Induce Penile Morphological Alterations in a Rat Model. Acta Cir. Bras. 29, 39–44. 10.1590/s0102-86502014001300008 25185055

[B30] Melman A. Bar-Chama N. McCullough A. Davies K. Christ G. (2006). hMaxi-K Gene Transfer in Males with Erectile Dysfunction: Results of the First Human Trial. Hum. Gene Ther. 17, 1165–1176. 10.1089/hum.2006.17.1165 17134370

[B31] Mills T. M. Chitaley K. Lewis R. W. (2001). Vasoconstrictors in Erectile Physiology. Int. J. Impot. Res. 13, S29–S34. 10.1038/sj.ijir.3900774 11781744

[B32] Molderings G. J. Van Ahlen H. Gothert M. Porst H. (1992). Modulation of Noradrenaline Release in Human Corpus Cavernosum by Presynaptic Prostaglandin Receptors. Int. J. Impot. Res. 4, 19–25.

[B33] Moreira E. D. Lbo C. F. Diament A. Nicolosi A. Glasser D. B. (2003). Incidence of Erectile Dysfunction in Men 40 to 69 Years Old: Results from a Population-Based Cohort Study in Brazil. Urology 61, 431–436. 10.1016/s0090-4295(02)02158-1 12597962

[B34] Moreland R. B. Kim N. Nehra A. Goldstein I. Traish A. (2003). Functional Prostaglandin E (EP) Receptors in Human Penile Corpus Cavernosum. Int. J. Impot. Res. 15, 362–368. 10.1038/sj.ijir.3901042 14562138

[B35] Murthy K. S. (2006). Signaling for Contraction and Relaxation in Smooth Muscle of the Gut. Annu. Rev. Physiol. 68, 345–374. 10.1146/annurev.physiol.68.040504.094707 16460276

[B36] Nicolosi A. Moreira E. D. Shirai M. Bin Mohd Tambi M. I. Glasser D. B. (2003). Epidemiology of Erectile Dysfunction in Four Countries: Cross-National Study of the Prevalence and Correlates of Erectile Dysfunction. Urology 61, 201–206. 10.1016/s0090-4295(02)02102-7 12559296

[B37] NIH (1993). Travelers' Diarrhea. National Institutes of Health Consensus Development Conference Statement. Natl. Inst. Health Consens Dev. Conf. Consens Statement 5, 1–7. 3892273

[B38] Porst H. (1996). The Rationale for Prostaglandin E1 in Erectile Failure: a Survey of Worldwide Experience. J. Urol. 155, 802–815. 10.1016/s0022-5347(01)66315-8 8583582

[B39] Rembold C. M. (1996). “Electromechanical and Pharmacomechanical Coupling,” in Biochemistry of Smooth Contraction. Editor Bárány M. (San Diego: Academic Press), 227–239. 10.1016/b978-012078160-7/50021-4

[B40] Saenz de Tejada I. Carson M. P. De Las Morenas A. Goldstein I. Traish A. M. (1991). Endothelin: Localization, Synthesis, Activity, and Receptor Types in Human Penile Corpus Cavernosum. Am. J. Physiol. 261, H1078–H1085. 10.1152/ajpheart.1991.261.4.H1078 1656784

[B41] Sanofi-Avenis (2010). “Randomized, Double-Blind, Placebo and Active Controlled Study of the Activity of SAR407899 Single-Dose on the Ability to Increase Duration of Penile Rigidity, under Experimental Condition,” in Patients with Mild to Moderate Erectile Dysfunction (Bethesda (MD): National Library of Medicine: ClinicalTrials.gov).

[B42] Schouten B. W. Bosch J. L. Bernsen R. M. Blanker M. H. Thomas S. Bohnen A. M. (2005). Incidence Rates of Erectile Dysfunction in the Dutch General Population. Effects of Definition, Clinical Relevance and Duration of Follow-Up in the Krimpen Study. Int. J. Impot. Res. 17, 58–62. 10.1038/sj.ijir.3901264 15510192

[B43] Shah J. (2002). Erectile Dysfunction through the Ages. BJU Int. 90, 433–441. 10.1046/j.1464-410x.2002.02911.x 12175404

[B44] Smith G. E. (1974). Papyrus Ebers. English Translation. Chicago: Ares Publishers.

[B45] Somlyo A. P. Somlyo A. V. (2003). Ca2+ Sensitivity of Smooth Muscle and Nonmuscle Myosin II: Modulated by G Proteins, Kinases, and Myosin Phosphatase. Physiol. Rev. 83, 1325–1358. 10.1152/physrev.00023.2003 14506307

[B46] Sommer F. Klotz T. Steinritz D. Schmidt A. Addicks K. Engelmann U. (2002). MAP Kinase 1/2 (Erk 1/2) and Serine/threonine Specific Protein Kinase Akt/PKB Expression and Activity in the Human Corpus Cavernosum. Int. J. Impot. Res. 14, 217–225. 10.1038/sj.ijir.3900856 12152110

[B47] Thomas J. A. (2002). Pharmacological Aspects of Erectile Dysfunction. Jpn. J. Pharmacol. 89, 101–112. 10.1254/jjp.89.101 12120751

[B48] Uckert S. Mayer M. E. Stief C. G. Jonas U. (2007). The Future of the Oral Pharmacotherapy of Male Erectile Dysfunction: Things to Come. Expert Opin. Emerg. Drugs 12, 219–228. 10.1517/14728214.12.2.219 17604498

[B49] Virag R. Shoukry K. Floresco J. Nollet F. Greco E. (1991). Intracavernous Self-Injection of Vasoactive Drugs in the Treatment of Impotence: 8-year Experience with 615 Cases. J. Urol. 145, 287–292. 10.1016/s0022-5347(17)38316-7 1671107

[B50] Webb R. C. (2003). Smooth Muscle Contraction and Relaxation. Adv. Physiol. Educ. 27, 201–206. 10.1152/advan.00025.2003 14627618

[B51] Woodrum D. A. Brophy C. M. (2001). The Paradox of Smooth Muscle Physiology. Mol. Cel Endocrinol. 177, 135–143. 10.1016/s0303-7207(01)00407-5 11377829

[B52] Yafi F. A. Jenkins L. Albersen M. Corona G. Isidori A. M. Goldfarb S. (2016). Erectile Dysfunction. Nat. Rev. Dis. Primers 2, 16003. 10.1038/nrdp.2016.3 27188339 PMC5027992

[B53] Zelefsky M. J. Shasha D. Branco R. D. Kollmeier M. Baser R. E. Pei X. (2014). Prophylactic Sildenafil Citrate Improves Select Aspects of Sexual Function in Men Treated with Radiotherapy for Prostate Cancer. J. .Urol. 192, 868–874. 10.1016/j.juro.2014.02.097 24603102

